# Forensic facial examiners versus super-recognizers: Evaluating behavior beyond accuracy

**DOI:** 10.1002/acp.4003

**Published:** 2022

**Authors:** Carina A. Hahn, Liansheng Larry Tang, Amy N. Yates, P. Jonathon Phillips

**Affiliations:** 1 Information Access Division, National Institute of Standards and Technology, Gaithersburg, Maryland, USA; 2 Statistics and Data Science, University of Central Florida, Orlando, Florida, USA

**Keywords:** facial forensics, facial identification, facial proficiency, forensic facial examiner, super-recognizer

## Abstract

We evaluated the detailed, behavioral properties of face matching performance in two specialist groups: forensic facial examiners and super-recognizers. Both groups compare faces to determine identity with high accuracy and outperform the general population. Typically, facial examiners are highly trained; super-recognizers rely on natural ability. We found distinct behaviors between these two groups. Examiners used the full 7-point identity judgment scale (−3: “different”; +3: “same”). Super-recognizers’ judgments clustered toward highly confident decisions. Examiners’ judgments for same- and different-identities were symmetric across the scale midpoint (0); super-recognizers’ judgments were not. Examiners showed higher identity judgment agreement than super-recognizers. Despite these qualitative differences, both groups showed insight into their own accuracy: more confident people and those who rated the task to be easier tended to be more accurate. Altogether, we show to better understand and interpret judgments according to the nature of someone’s facial expertise, evaluations should assess more than accuracy.

## 1 | INTRODUCTION

Stories of super-recognizers never forgetting a face and forensic facial examiners identifying criminals have sparked the interest of researchers, the press, and the public worldwide. Facial examiners’ decisions are trusted to the degree that they can testify in court—partly due to the expectation that they receive extensive training and mentoring (FISWG, 2019, 2020). Whereas super-recognizers have an innate ability for face recognition, typically demonstrated by achieving a high score on a facial recognition test (Noyes et al., 2017). Because of their ability, super-recognizers are employed in law enforcement and security organizations worldwide (Bate, Frowd, et al., 2019; Davis et al., 2016; Noyes et al., 2017; Phillips et al., 2018). For both facial examiners and super-recognizers, their high accuracy is well-established (Bate et al., 2018; Bate, Frowd, et al., 2019; Bate & Dudfield, 2019; Bobak, Bennetts, et al., 2016; Bobak, Dowsett, et al., 2016; Bobak et al., 2017; Bobak, Hancock, et al., 2016; Davis, Bretfelean, et al., 2019; Davis et al., 2016; Davis, Maigut, et al., 2019; Lee et al., 2009; Noyes, Davis, et al., 2021; Noyes et al., 2017; Phillips et al., 2018; Ramon et al., 2019; Robertson et al., 2016; Russell et al., 2009; Tardif et al., 2018; Towler et al., 2017; White et al., 2021; Wilkinson & Evans, 2009). What remains poorly understood are the cognitive underpinnings of their superior face matching ability. A recent synthesis of studies on facial expertise addresses these cognitive factors and converges on the idea that there are two cognitive routes to achieve facial expertise (Towler et al., 2021). However, this question has yet to be directly addressed within a single experimental study.

Prior studies which compared the accuracy and qualitative behavioral properties of facial examiners or super-recognizers have done so separately (Bate, Bennetts, et al., 2019; Bate et al., 2018; Bate, Frowd, et al., 2019; Bate & Dudfield, 2019; Bobak, Bennetts, et al., 2016; Bobak, Dowsett, et al., 2016; Bobak et al., 2017; Bobak, Hancock, et al., 2016; Davis, Bretfelean, et al., 2019; Davis et al., 2016; Davis, Maigut, et al., 2019; Hu et al., 2017; Norell et al., 2015; Noyes et al., 2017; Noyes, Parde, et al., 2021; Ramon et al., 2019; Robertson et al., 2016, 2019; Russell et al., 2009; Tardif et al., 2018; Towler et al., 2017, 2021; White et al., 2015; Wilkinson & Evans, 2009). In the only experiment which directly compared facial examiners to super-recognizers, the authors found they were equally accurate to each other (Phillips et al., 2018). This opens the questions: are facial examiners a subset of super-recognizers, with training and credentials to testify in legal proceedings? If not, how are they different, and are those differences relevant to their professional duties? By moving beyond accuracy alone, we can study these questions. The answers will inform how to effectively measure facial proficiency and how to interpret judgments from examiners and super-recognizers. Our results point to the need for a more comprehensive assessment of facial recognition ability. In our study, we expanded on data from a prior study (Phillips et al., 2018) to harness the unique opportunity to juxtapose these highly, and equally, accurate groups whose facial expertise differs in nature. This allowed us to evaluate how they are the same and how they are different, independent of overall accuracy. We moved beyond accuracy to investigate the properties of facial examiners’ and super-recognizers’ facial comparison performance.

We build on Phillips et al. (2018) which assessed the accuracy of facial examiners, super-recognizers, and control groups. For each facial comparison, participants viewed two images. Some comparisons depicted the same identity; others depicted different identities. Participants provided two responses for each comparison: an identity judgment and a difficulty rating. The study focused on individual accuracy and characterized groups based on those differences. Accuracy was measured using area under the receiver operating characteristic curve (AUC). For facial examiners, the median AUC was 0.93; for super-recognizers, the median AUC was 0.83. These were statistically equivalent as measured with a Mann–Whitney U test (U = 331, *p* = .56).

In the current study, we used these previously collected data to examine what the distribution of identity judgments looked like along a 7-point identity judgment scale (range: −3: “The observations strongly support that this is not the same person” to +3: “The observations strongly support that it is the same person”). We assessed the consistency of identity judgments across participants. Ideally, the same facial comparison will result in the same conclusion across individuals. This is important for confidence in the facial comparison process. Finally, we examined how identity judgments and difficulty ratings correlated and how these measures correlated with a person’s own accuracy to assess metacognitive ability.

These findings inform whether these groups are the same, behaviorally and cognitively; if different strategies can underpin equal accuracy; and whether the nature of one’s expertise affects performance. These are essential to effectively measure multiple aspects of facial proficiency. On an applied level, these findings support that the nature of one’s expertise is related to different underlying behaviors. In our study, to qualify as a super-recognizer, the participant could never have received any forensic training. The facial examiners in our study did receive training. Therefore, a history of training (or lack of training) was a defining difference in the backgrounds of facial examiners and super-recognizers. The role of training and/or professional experience could not be tested definitively in this study, but our conclusions suggest that the behavioral properties of examiners are consistent with the goals of forensic training. Usually, the effect of training is measured by changes in accuracy (Towler et al., 2019). This work shows that the effect of training and/or professional experience may be assessed with other desired measures of training success. By directly measuring behaviors of interest, we can inform training programs to support forensic applications.

On a theoretical level, these results show that rather than a single strategy supporting high accuracy, multiple strategies can lead to highly accurate at face matching. Investigating these underlying behaviors reveals how the nature of facial expertise can vary depending on one’s background. From this, we can begin teasing apart the cognitive processes supporting superior facial comparison ability.

## 2 | METHODS

### 2.1 | Procedure

**FIGURE 1:**
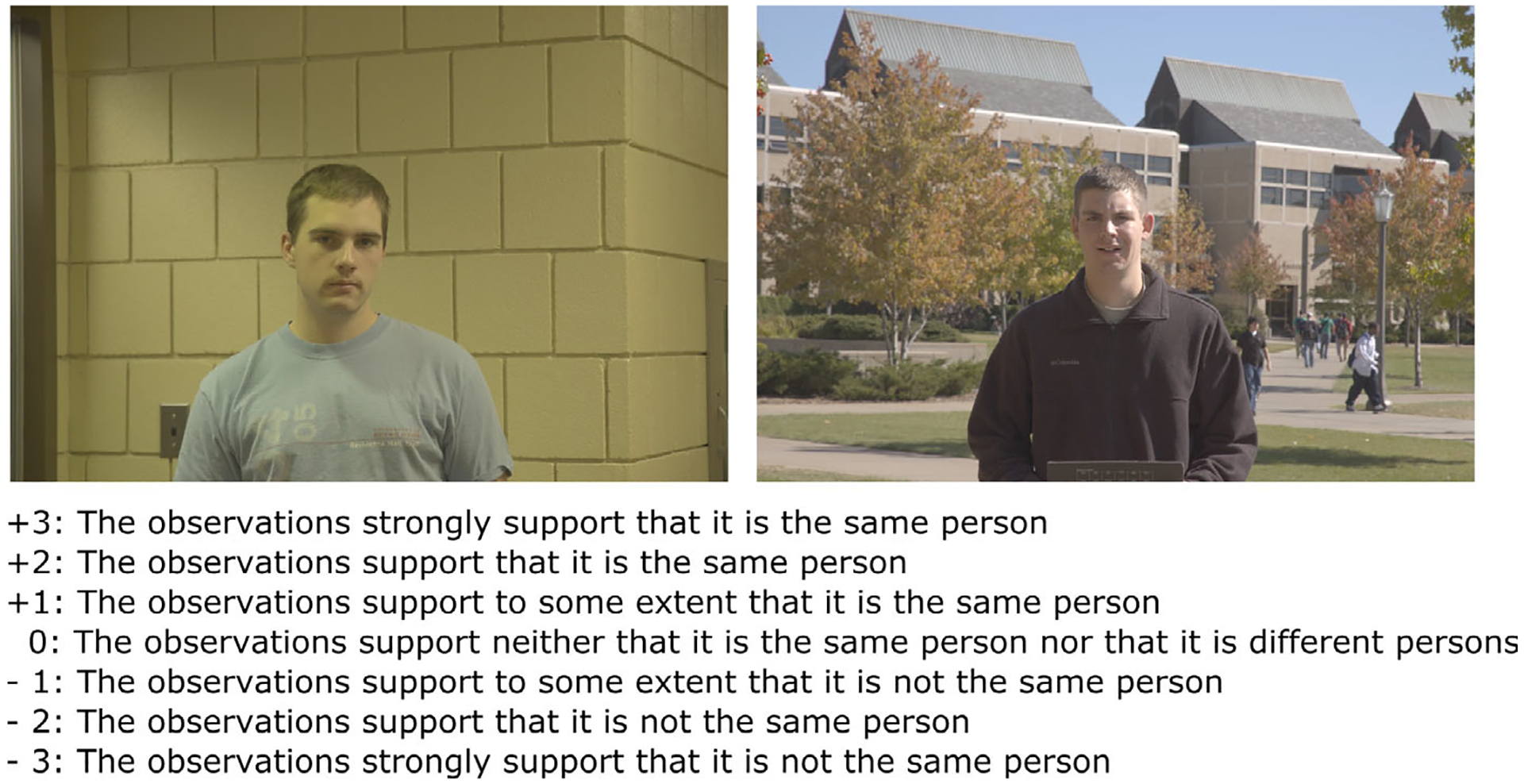
An example facial comparison and the identity judgment scale from this study. Participants viewed pairs of faces and were instructed to determine identity on the 7-point scale shown above. For each comparison, participants also provided a difficulty rating (not shown in the figure). Facial examiners and super-recognizers were allowed 3 months to submit their responses and could use any tools and methods they had available. All images from the study and correct answers can be viewed in the supplementary information for Phillips et al. (2018)

On each trial, participants compared two facial images and determined whether they depicted the same identity or different identities. Figure 1 shows an example comparison and the full identity judgment scale. There were 12 same-identity and eight different-identity trials in the task. This imbalance prevented participants from applying a process of elimination strategy to determine identity. All face images were sent electronically to the participants. One of the objectives in the original study was to measure facial comparison ability under conditions that mirrored real-world casework. To accomplish this, facial examiners and super-recognizers were allotted up to 3 months to submit their responses, and they were able to use any tools and methods of their choice when completing the task. This allowed them to perform the task under their preferred viewing and working conditions, using their tools and methods. Comparisons could be completed in any order.

Identity judgments were obtained using a 7-point identity judgment scale (−3: the observations strongly support that it is not the same person; −2: the observations support that it is not the same person; −1: the observations support to some extent that it is not the same person; 0: the observations support neither that it is the same person nor that it is different persons; +1: the observations support to some extent that it is the same person; +2: the observations support that it is the same person; +3: the observations strongly support that it is the same person). This identity judgment scale was developed in consultation with members of the forensic face community and is based on scales used in forensic work (e.g., see Conclusion Scale from Norell et al. (2015)). These identity judgments were the basis of their accuracy, using the area under the receiver operating characteristic curve (AUC).

In addition to providing an identity judgment, participants rated the difficulty of the comparison on a 5-point difficulty rating scale (1: Easy, The comparison was easier than most facial comparisons; 2: Moderate, The comparison was a typical facial comparison; 3: Difficult, The comparison was more difficult than most facial comparisons; 4: Very Difficult, The comparison was unusually difficult, involving significant photometric illumination, or pose changes, other red flags; 5: Not Possible, The comparison was virtually impossible, due to a lack of detail in the image(s)). This was included to evaluate if the perceived difficulty of each facial comparison provided independent information from the identity judgment.

### 2.2 | Stimuli

Images are covered under the license for the Forensic Facial Examiner Study Data Set from the University of Notre Dame available from https://cvrl.nd.edu/projects/data/\#forensic-facial-examiner-study-data-set. All facial comparisons in this study appear in the supplemental materials of the original study from which the data was obtained (Phillips et al., 2018).

### 2.3 | Participants

In the study from which this data was obtained (Phillips et al., 2018), the authors measured the accuracy of forensic facial examiners, forensic facial reviewers, super-recognizers, fingerprint examiners, under-graduate students, and face-recognition algorithms on a facial comparison task. In the current study, we examined a subset of these participant groups. We compared two highly skilled face specialist groups: forensic facial examiners, or facial examiners, (*n* = 57, 28 females) and super-recognizers (*n* = 13, 8 females). Facial examiners were recruited through emails to the Facial Identification Scientific Working Group (FISWG) and the Organization of Scientific Area Committees for Forensic Science, Facial Identification Subcommittee (OSAC FI Subcommittee). Super-recognizers were recruited by email from a participant pool of a different study (see supplementary information for Phillips et al., 2018). Facial examiners were defined as those who performed one-to-one facial comparisons as part of their profession. In these types of facial comparisons, only two images are evaluated at a time (as opposed to comparing a face to a gallery of images to judge identity). Super-recognizers were defined as those actively employed as a super-recognizer, or who had completed a face recognition test which categorized them as a super-recognizer (e.g., common tests used to assess super-recognizer performance as reviewed in (Dunn et al., 2020; Noyes & O’Toole, 2017; Ramon, 2021)). Super-recognizers in this study never received any forensic comparison training. Because of this, no one could qualify as both a facial examiner and super-recognizer.

The National Institute of Standards and Technology Institutional Review Board reviewed and approved the protocol for this project and all subjects provided informed consent in accordance with 15 CFR 27, the Common Rule for the Protection of Human Subjects.

### 2.4 | Statistical analysis

Final analyses and visualizations were completed using R version 4.2.0 and corresponding packages (Bengtsson, 2021; Csárdi & FitzJohn, 2019; Gwet, 2014a; Kassambara, 2020; Lawrence, 2016; Mangiafico, 2021; Neuwirth, 2014; R Core Team, 2021; Selker et al., 2021; Wickham, 2019) and executed in RStudio version 2021.09.0 (RStudio Team, 2021). Code is available from https://github.com/usnistgov/face-recognition-humans-machines.

Accuracy was measured using the area under the receiver operating characteristic curve (AUC) for each individual. The inputs to compute the AUC were the identity judgments themselves, labeled by trial type. Confidence was defined as the absolute value of an identity judgment. See Methods Section, Experimental Design for the full identity judgment scale. This yielded a possible range of 0–3. Difficulty ratings were the responses on the difficulty rating scale.

Each section of results contains its pertinent methodological information. In what follows, we expand on select analyses for clarity and replicability.

#### 2.4.1 | Identity judgment distributions

**FIGURE 2:**
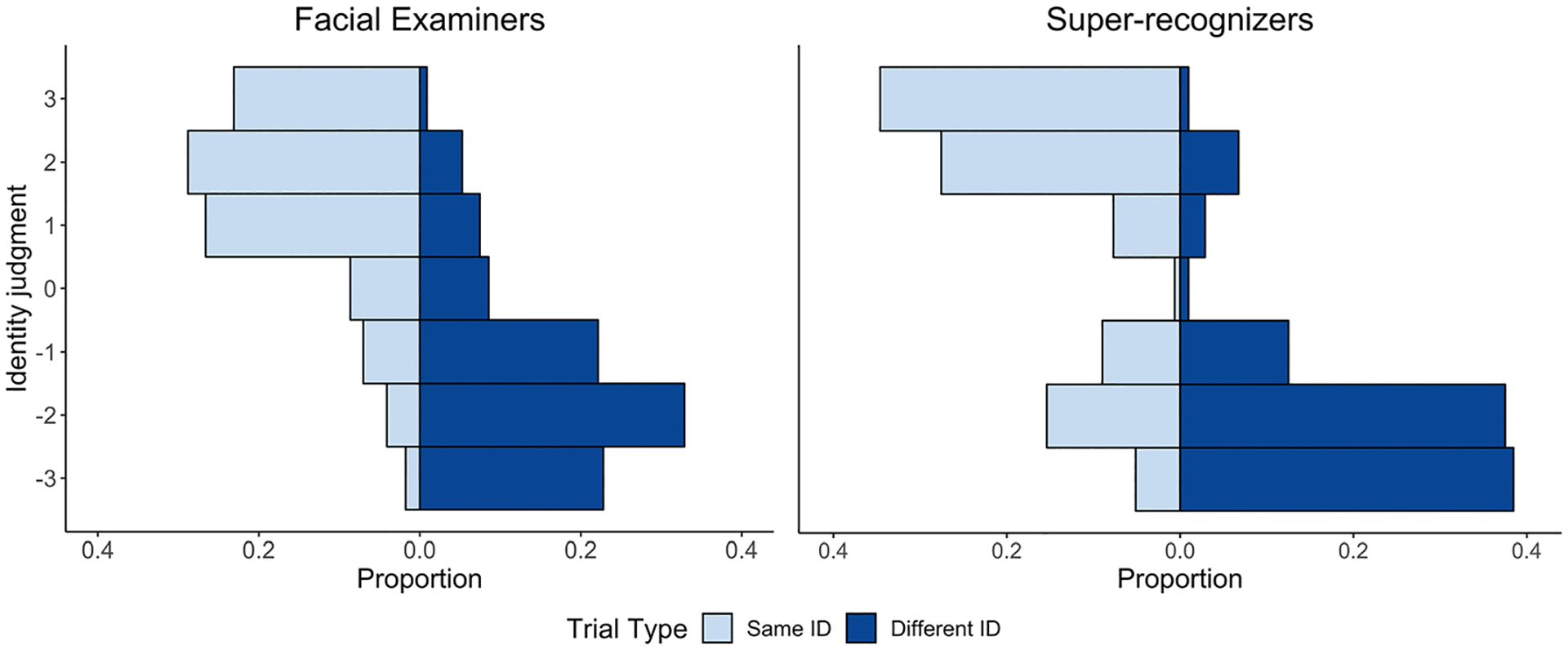
Back-to-back histograms depicting the proportion of judgments (x-axis) of a given identity judgment (y-axis). Facial examiners are shown in the left-side graph; super-recognizers on the right-side graph. For each graph, the left, light blue side shows the response distributions for same-identity trials. The right, dark blue side shows the distributions for different-identity trials

From all identity judgments provided in the test, we produced a distribution of the responses to reflect the frequency of each judgment. First, we computed the number of judgments associated with a given point on the scale relative to the total number of trials, separately for same- and different-identity trials and for each participant group. The result of this was the proportion of responses associated with each scale option across all participants within a participant group and trial type (Figure 2). For statistical comparisons, we converted the frequencies of raw identity judgments into cumulative response distributions. These cumulative distributions were submitted to Kolmogorov–Smirnov (K-S) tests. For each distribution comparison, we conducted a two-sample Kolmogorov–Smirnov (K-S) test between the two distributions of interest. We conducted four comparisons in total: (1) facial examiners versus super-recognizers for same-identity trials, (2) facial examiners versus super-recognizers for different-identity trials, (3) same- versus different-identity trials within facial examiners, and (4) same- versus different identity trials within super-recognizers. To compare the frequency of judgments across same- and different-identity trials within each participant group, the judgments associated with different-identity trials were reversed, or *mirrored*, such that responses of −3 were coded as +3, and vice versa. This reverse coded different-identity distribution was submitted to the analysis and compared to the same-identity distribution.

To compare the frequencies of responses at the scale extremes (±3) and the scale midpoint (0), we computed the frequency of each judgment for each participant relative to the total number of trials. For each participant, this gives us the proportion of judgments corresponding to a particular decision. Statistical comparisons were conducted with non-parametric, Mann–Whitney U tests with the effect size, r, defined as:

$$r=Z/\sqrt{N},$$

where *Z* is the *Z* score of the test statistic, and *N* is the number of observations

#### 2.4.2 | Identity judgment agreement

To evaluate the consistency of responses across facial examiners and super-recognizers, we measured the inter-rater reliability of the identity judgments across all possible unique pairs of individuals within each participant group. For facial examiners, this produced *n* = 1596 unique pairs of participants. For super-recognizers, this produced *n* = 78 unique pairs of participants. Inter-rater reliability was computed using Cohen’s Kappa, weighted for ordinal data $\left({\hat{k}}_{c}^{'}\right)$ (Cohen, 1968b; Gwet, 2014b).

We tested for group differences with a bootstrap resampling procedure (e.g., Harden (2011)). Details are available in the results section for this analysis.

## 3 | RESULTS

### 3.1 | Identity judgment distributions

We started with the basic question: do facial examiners and super-recognizers use the identity judgment scale differently? Visually inspecting the identity judgment distributions indicates that, yes, judgments between the two groups varied (Figure 2). Facial examiners’ judgments were distributed across the full scale. Super-recognizers, on the other hand, selected the scale extremes and avoided the midpoint 0 (“the observations support neither that it is the same person nor that it is different persons”). This was reflected in the modes of the distributions. The mode judgments for facial examiners were +2 and −2 for same- and different-identity trials, respectively. Super-recognizers, by contrast, selected +3 and −3 most frequently for same-identity and different-identity trials, respectively.^i^

Subsequent statistical analyses supported these descriptive observations and visual inferences. For statistical analysis, we compared the distribution of responses from facial examiners and super-recognizers with two-sample Kolmogorov–Smirnov (K-S) tests. We conducted two, separate tests for same- and different-identity trials. We found that for both trial types, facial examiners and super-recognizers used the scale differently (same-identity trials: *D* = 0.17, *p* = .002; different-identity trials: *D* = 0.20, *p* = .002).

The second basic question is whether participants in each group approached the scale similarly for the two trial types. As stated, the absolute values of the identity judgments were the same for each trial type, within a given group (facial examiners: +2 or −2; super-recognizers: +3 or −3). However, the overall distributions paint a more thorough picture of their response patterns. Based on a visual inspection of Figure 2, the same- and different-identity distributions appeared to be mirrored for facial examiners. This suggests they approach the scale similarly for comparisons of same- and different-identities. However, for super-recognizers, this mirroring was less apparent. To test this statistically, we mirrored different-identity judgments to reflect the direction of same-identity judgments by reversing the scale direction for different-identity trials (e.g., −3 was converted to +3 and vice versa). We then compared the distributions from same- and mirrored different-identity judgments (Figure S2). This process was applied to both participant groups. For facial examiners, the cumulative distributions were almost completely overlapping. This indicates no difference between their scale use for same- and different-identity trials (K-S test: *D* = 0.04, *p* = 0.82). For super-recognizers, confidence increased more sharply for same-identity trials compared to different-identity ones (K-S test: *D* = 0.19, *p* = 0.02). By characterizing the full response scale, this indicates that super-recognizers are more cautious overall on different-identity trials.

Therefore, equal accuracy was supported by different approaches to the identity judgment scale. We next tested whether these differences appeared in other identity judgments properties: agreement, the relationship between different behavioral measures, and insight into accuracy.

### 3.2 | Identity judgment agreement

In forensic analysis, reliability is desired. Ideally multiple examiners comparing the same image pair will provide the same identity judgment – they will provide consistent answers. Measuring the consistency of judgments assesses the degree to which this ideal is met. To test the extent of agreement, we measured inter-rater reliability of the identity judgments. We modeled the case in which comparisons are completed independently by two examiners. To provide a bench-mark, we repeated the analysis with super-recognizers.

**FIGURE 3:**
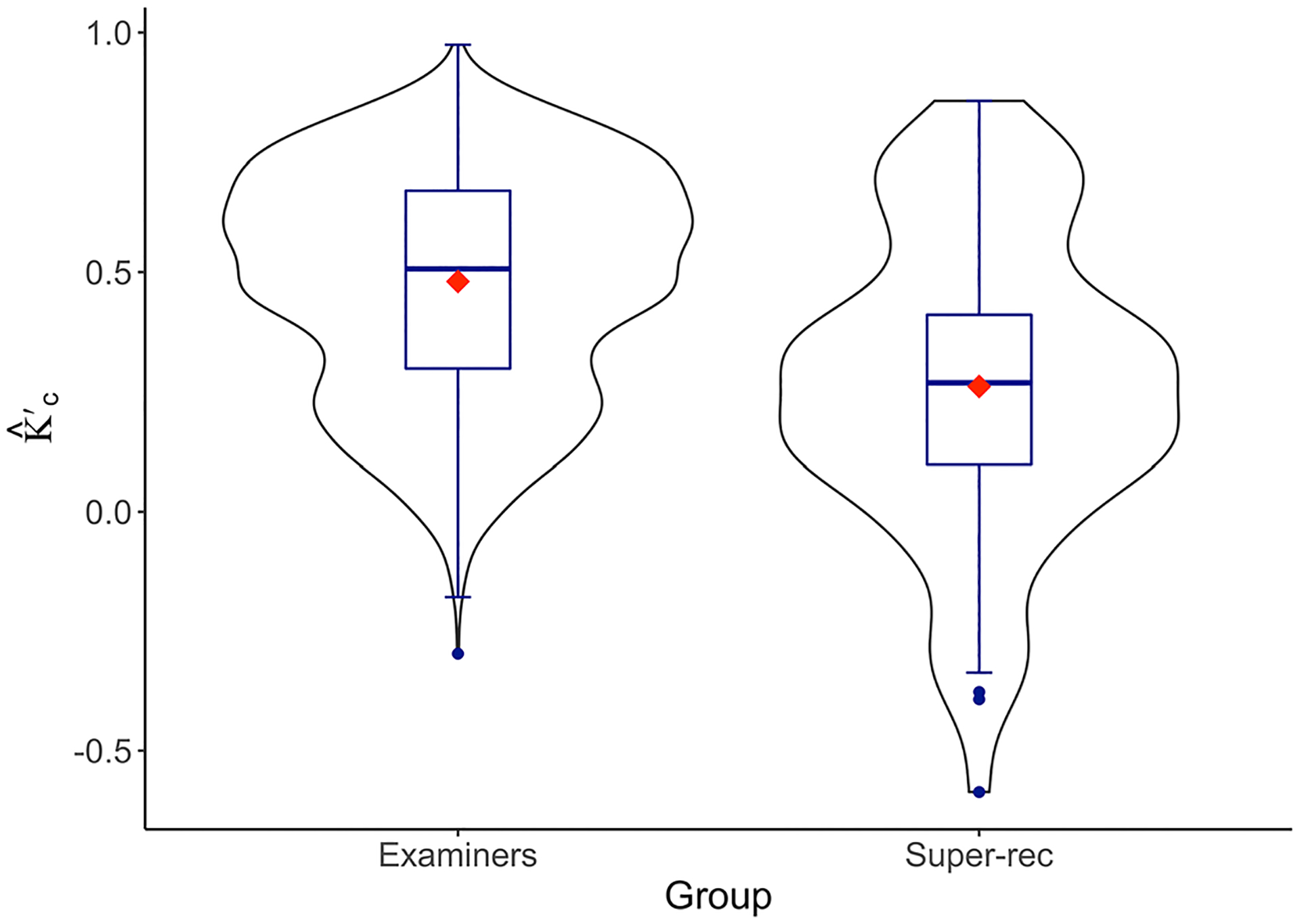
The distribution of agreement between all possible pairs of participants within each group. This is a violin plot with a box-and-whisker plot overlay. The violin plot represents the range and density of different levels of agreement ${\hat{k}}_{c}^{'}$ A box-and-whisker plot overlay shows the median, interquartile ranges, and the full range of agreement for each group. Points represent outliers. Red diamonds show the mean for each group.

We measured identity judgment agreement on all 20 facial comparisons separately for each participant group. Agreement was assessed using a standard measure of inter-rater reliability, Cohen’s Kappa, weighted for ordinal data ${\hat{k}}_{c}^{'}$ (Cohen, 1968a; Fleiss et al., 2003; Gwet, 2014b). The ordinal weighting was applied to reflect the identity judgment response scale. The possible range of ${\hat{k}}_{c}^{'}$ between two participants was +1 (indicating perfect agreement) to −1 (indicating complete disagreement). Agreement was measured between all possible unique pairs of facial examiners. With 57 facial examiners in our sample, this produced 1596 ${\hat{k}}_{c}^{'}$ values. Between the 13 super-recognizers in our study, there were 78 unique pairs of participants and corresponding ${\hat{k}}_{c}^{'}$.values (Figure 3).

Average pairwise agreement was nominally higher among facial examiners (*M* = 0.48, SD = 0.24) compared to agreement among super-recognizers (*M* = 0.26, SD = 0.32). Rule of thumbs from (Fleiss et al., 2003) suggest that ${\hat{k}}_{c}^{'}$ 0.75 indicates excellent agreement; ${\hat{k}}_{c}^{'}$ between 0.75 and 0.40 indicates fair agreement, and ${\hat{k}}_{c}^{'}$ 0.40 indicates low agreement. Although context guides these interpretations, this rule of thumb suggests facial examiners show a fair amount of agreement, and super-recognizers show poor agreement.

We tested whether the observed agreement between facial examiners and super-recognizers was statistically significant. Because ${\hat{k}}_{c}^{'}$ values were measured between all possible pairs of participants within a group, each person contributed to multiple ${\hat{k}}_{c}^{'}$ values. This lack of independence between measures precluded the use of standard statistical tests because the variance of the statistic is not readily available for such dependent measures. Therefore, we implemented a bootstrap resampling procedure (e.g., Harden (2011)) to compare the two groups. For each bootstrap iteration, 20 trials (facial comparisons) were selected randomly with replacement to replicate the number of trials presented in the original experiment. For these selected trials, we measured ${\hat{k}}_{c}^{'}$ between all possible, unique pairs of individuals within each participant group, repeating the original procedure described above. Next, we computed the difference between the average ${\hat{k}}_{c}^{'}$ values for facial examiners and for super-recognizers on that iteration. We refer to this as the ${\hat{k}}_{c}^{'}$ difference. We repeated this process for 1000 iterations which produced a distribution of 1000 ${\hat{k}}_{c}^{'}$ differences between the two groups (see Figure S3). We determined if agreement was significantly different between the two groups according to a 95% bootstrap confidence interval (CI) around the top and bottom 2.5% of the distribution. If the 95% CI did not intersect 0 (indicating no difference), the differences between the two groups were judged to be statistically significant.

The 95% CI for this distribution of ${\hat{k}}_{c}^{'}$ differences was (0.08, 0.35). This indicates that agreement differed significantly across the two groups. While the lower bound of this CI determined significance, the upper bound characterized the probable range of agreement differences between the two groups. Therefore, with 95% confidence, our estimate of the agreement difference range between examiners and super-recognizers extended from a small (0.08) to a large difference (0.35).

### 3.3 | Relationship between behavioral measures

The previous analyses focused on differences in identity judgment scale use and agreement between facial examiners and super-recognizers. Next, we conducted a deeper analysis into the behavioral measures obtained in this task: the confidence of the identity judgments and difficulty ratings. This provides insight into the shared and unique information across different behavioral measures. We collected both an identity judgment on a 7-point scale and a difficulty rating for each trial (see Section 3). We obtained confidence from the absolute value of the identity judgment. For example, a judgment of +3 or −3 implied high confidence. We expect that a high confidence judgment would be associated with a lower difficulty rating. Likewise, low absolute values in the identity judgment (i.e., 0) implied low confidence, and we predicted that these decisions would be associated with greater rated difficulty. By measuring the extent to which these are related, we addressed the question of whether these ratings provided distinct or overlapping information. Note that confidence does not necessarily indicate accuracy: a judgment might be highly confident but erroneous.

Considering the accuracy of judgments was the second part of this analysis. Identity judgments were the basis of accuracy as measured by the area under the receiver operating characteristic curve (AUC). We measured metacognitive ability: the extent to which people were aware of their own ability. To examine the level of this behavioral insight, we tested whether accuracy was associated with confidence and rated difficulty.

#### 3.3.1 | Confidence and difficulty

We evaluated the relationship between rated difficulty and confidence at two levels of analyses: (1) at the level of the trials themselves to address the question: how much does the rated difficulty of a facial comparison correspond to the confidence in that identity judgment? and (2) at the level of the individual to address: does a person who rated the task to be more difficult overall also respond with less confidence overall? The effect of rated difficulty on performance itself has been well-studied (see Scasserra (2008) for a review). However, to our knowledge, the relationship between the rated difficulty and confidence of a given response has not been studied. For this analysis, we only considered the confidence itself, not the accuracy of the judgment.

**FIGURE 4:**
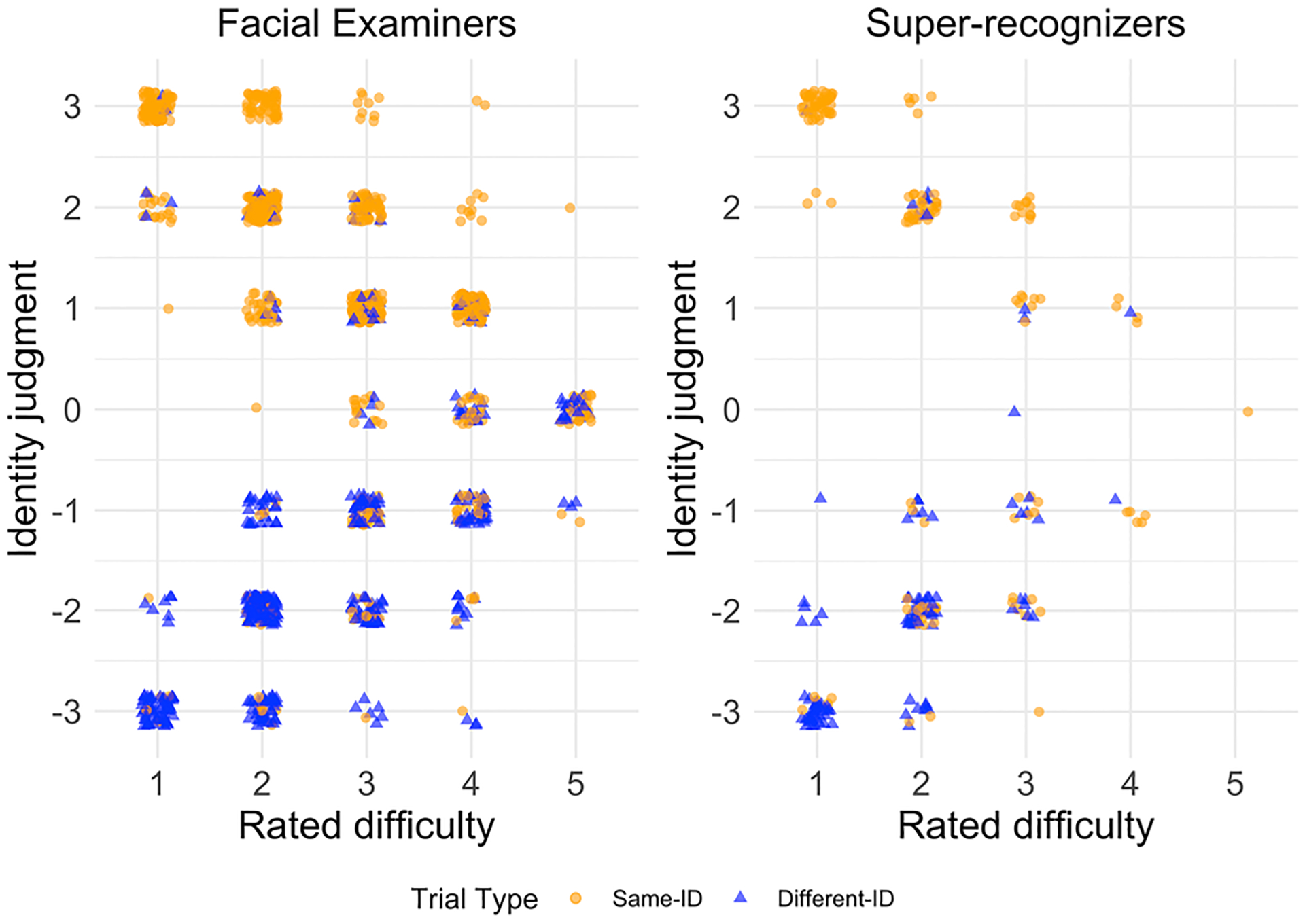
Two scatterplots showing the relationship between difficulty ratings and identity judgments. Facial examiners are shown on left-side graph; super-recognizers on the right-side graph. Each point represents one judgment. Point color and shape indicate trial type. Points are jittered at each grid intersection to show overlapping points. For identity judgments, responses with lower absolute values indicated lower confidence; responses with higher absolute values indicated higher confidence. For difficulty ratings, lower values indicated greater ease than higher values

First, to analyze the relationship between difficulty ratings and confidence at the level of the trials themselves, we plotted every combination of difficulty rating and identity judgment observed across all trials (Figure 4). A visual inspection shows that as the difficulty rating increased, the confidence associated with the identity judgment decreased, for both correct and incorrect judgments. For example, there were no cases in which the lowest difficulty rating was provided (1) with an identity judgment at the midpoint (0). Likewise, there were no cases in which a high difficulty rating (5) was provided with a highly confident (+/–3) judgment.

Figure 4 shows all responses collected, one point per response. This gives a full visualization of all responses in the test. Our goal was to understand how the confidence of a response related to their rated difficulty, statistically. To obtain a pure measure of confidence, regardless of trial type or scale direction (positive or negative), we first converted the raw identity judgments to a measure of confidence (the absolute value of the identity judgment). This retained the confidence of the identity judgment regardless of the direction of the decision (i.e., judged to be the same identity or different identities). As a result, some incorrect decisions will get “flipped” (e.g., a judgment of −3 on same identity trials are evaluated the same as a +3.). Therefore, this analysis cannot infer anything about accuracy. It is purely a measure of how one’s confidence relates to their reported perception of trial difficulty. In a subsequent analysis, we account for accuracy relative to confidence (Figure 6). We examined the relationship between confidence and rated difficulty statistically with a Kendall’s t correlation. Results showed that as difficulty ratings increased, confidence decreased for both groups (facial examiners: Kendall’s τ = −0.67, *p* < .001; super-recognizers: τ = −0.76, *p* < .001).

**FIGURE 5:**
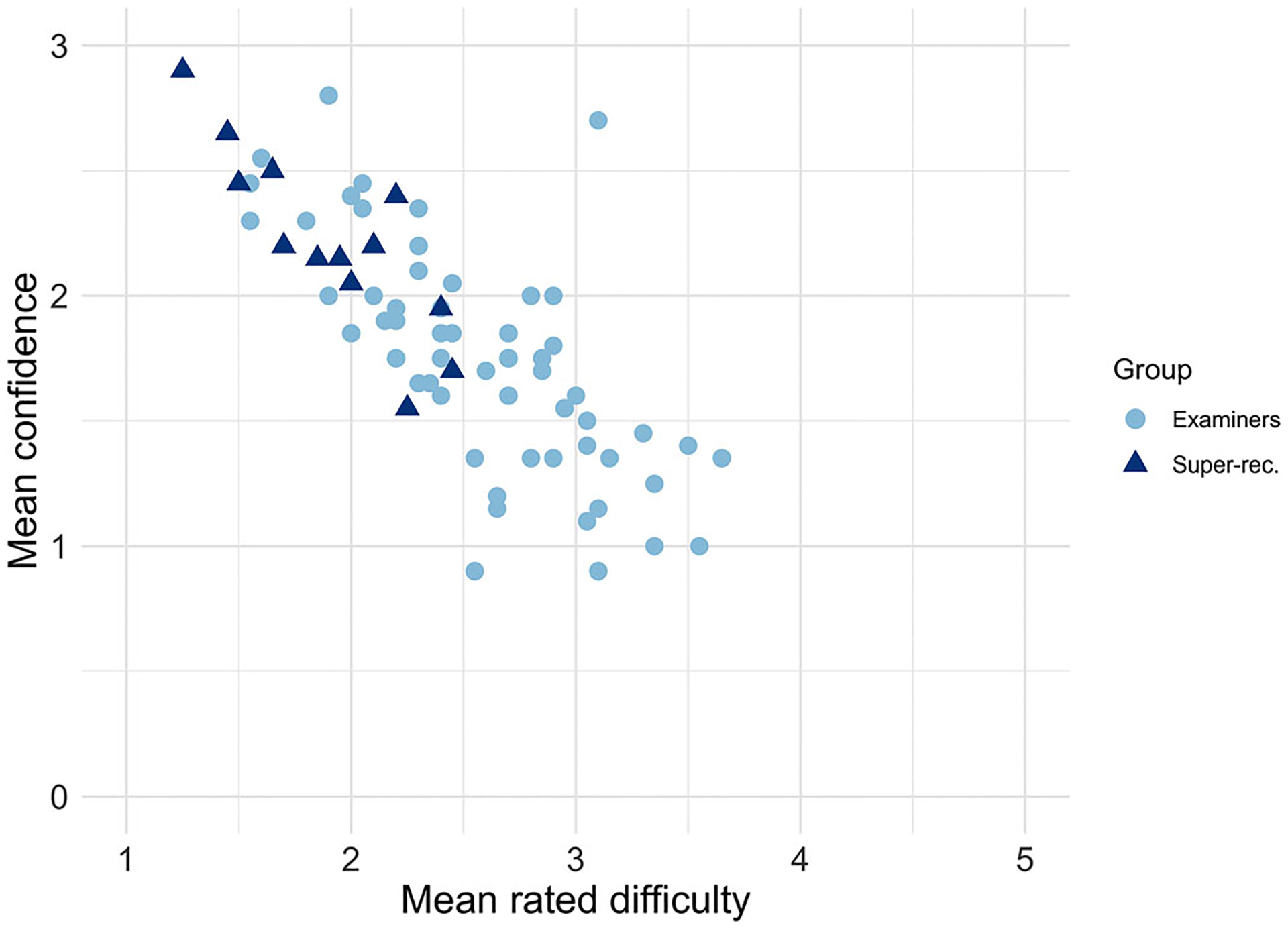
A scatterplot showing the relationship between rated difficulty and confidence at the level of the individuals. Each point represents one participant. A point’s position on the *x*-axis indicates a participant’s mean rated difficulty across the whole task. The position on the *y*-axis indicates the participant’s mean confidence across the task. Point color and shape indicate participant group: light blue circles represent facial examiners; dark blue triangles represent super-recognizers. For confidence, lower values indicate a lower level of confidence; higher values reflect higher confidence. For difficulty ratings, lower values indicate ease; higher values indicate greater difficulty.

This trial-based analysis provided a full-test view of the relationship between confidence and difficulty. However, this does not address whether people who are more confident rate the task to be less difficult. To test this, we computed each participant’s average confidence and difficulty rating across the whole task. We then computed the correlation between their average confidence and their average difficulty rating. For both groups, there was a negative correlation between confidence and difficulty: the more difficult they found the task, the lower their confidence (facial examiners: τ = −0.56, *p* < .001, super-recognizers: τ = −0.70, *p* = .001, Figure 5).

#### 3.3.2 | Behavioral insight: Confidence, difficulty, and accuracy

We tested the extent to which face specialists have insight into their own accuracy. Previous studies have measured the extent to which super-recognizers can estimate their own accuracy using self-report questionnaires to estimate face recognition ability (Bate &Dudfield, 2019; Bobak et al., 2018). Instead of self-estimated ability, our measure of insight was the degree to which a person’s confidence and difficulty ratings were correlated with their accuracy on the test. For each participant, we computed their mean confidence and difficulty rating across the entire task. Accuracy was measured using AUC. Detailed analyses of difficulty ratings can be found in the Supporting information.

**FIGURE 6:**
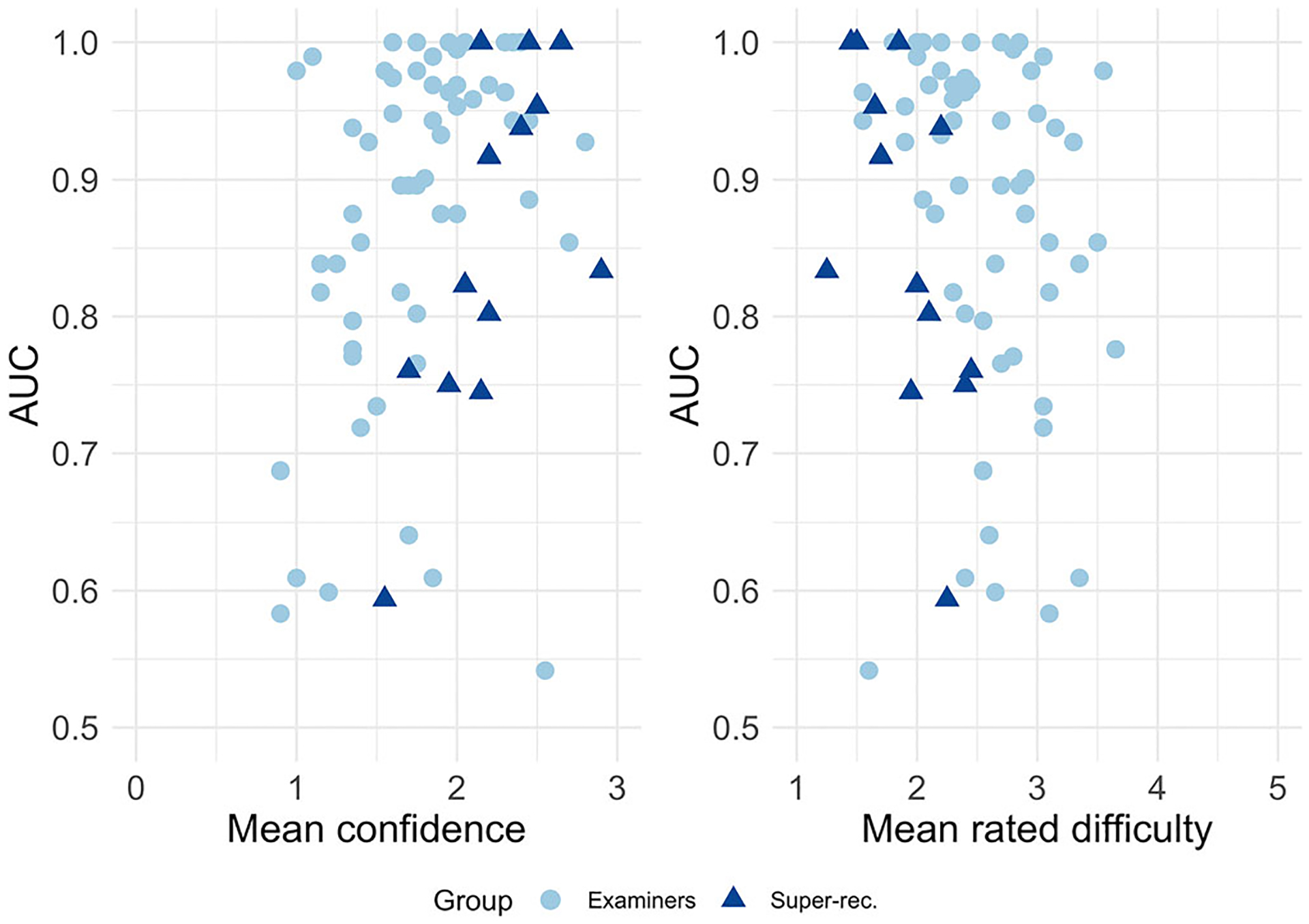
Two scatter plots show the relationship between behavioral measures and accuracy (AUC). Each point represents one participant. Point color and shape reflect participant group: light blue circles represent facial examiners; dark blue triangles represent super-recognizers. A point’s position on the *x*-axis indicates the participant’s mean confidence (left-side graph) or mean difficulty rating (right-side graph). Position on the *y*-axis indicates the participant’s accuracy. For confidence, lower values indicate a lower level of confidence; higher values reflect higher confidence. For difficulty ratings, lower values indicate ease; higher values indicate greater difficulty

First, we measured the relationship between participants’ mean confidence and accuracy with Kendall’s τ correlation. This was computed separately for facial examiners and super-recognizers. For both groups, participants who were more confident on average tended to be more accurate (facial examiners: τ = 0.29, *p* = .002; super-recognizers: τ = 0.54, *p* = .01, Figure 6). Therefore, those in both face specialist groups demonstrated insight into their own accuracy. The relationship between mean rated difficulty and accuracy was consistent with those of mean confidence: people who found the task more difficult overall tended to be less accurate (facial examiners: τ = −022, *p* = .02; super-recognizers: τ = −0.48, *p* = .023, Figure 6).

## 4 | DISCUSSION

Are facial examiners and super-recognizers the same? Our answer is: no. Our conclusion required investigating behaviors beyond accuracy. Identity judgment distributions and consistency differed. Super-recognizers preferred judgment scale extremes (+3/−3); facial examiners took advantage of the full identity judgment scale. Facial examiners agreed with each other to a greater extent than super-recognizers. Despite these differences, both groups showed metacognitive insight into their ability. These results provide insight into our cognitive understanding of facial expertise.

Others propose the “two-routes” hypothesis for facial expertise: one via training for facial examiners and another via natural ability for super-recognizers (Towler et al., 2021). Our work supports the existence of these two-routes by showing that facial examiners and super-recognizers have distinct behaviors. However, from the current bodies of work, we do not know the extent to which training influences behaviors. For example, if a super-recognizer takes training, and their scale use changes, this suggests behaviors may malleable. Otherwise, if behavior is fixed, this suggest that training filters for individuals who naturally have the behaviors desired for examiners.

Differences in scale use carry important, real-world implications. Law enforcement investigations and legal proceedings rely partly on the confidence of facial recognition judgments. A high confidence judgment could carry more weight than one with low confidence. Because super-recognizers have a higher tendency to use the most confident decision point, their judgments have different meanings than a highly confident decision from facial examiners. Interpreting a super-recognizer’s decision the same way as a facial examiner’s potentially result in miscarriages of justice. This suggests interpretations of identity judgments may need to be tailored to each group.

The scale use differences observed in the current study are consistent with those observed in (Phillips et al., 2018). In their study, they estimated the likelihood of high confidence errors. Super-recognizers were more likely to make high confidence errors on faces of the same identity compared to faces of different identities. Facial examiners were equally likely to make errors on each trial type (see Figure 5 of Phillips et al., 2018). In the currently study, we show this tendency holds when evaluating the full response scale: super-recognizers’ confidence increases more rapidly for same identity trials, even when incorrect.

For applications, perhaps the most important finding in this study was to show that although these two groups are equally accurate, facial examiners’ responses were more consistent. Ideally in forensic practice, an identity judgment would be based on the evidence itself – in this case, the similarity of the faces. By quantifying examiner agreement, we can better understand the degree to which this ideal occurs in practice. The scale in this study was developed in consultation with the forensic community to reflect those in their casework. It is possible that a response scale created with the super-recognizer community would have resulted in high consistency for that group. If so, this would suggest that training and/or experience plays a role in how the judgment scales themselves are interpreted by the person providing the judgment.

Facial examiners and super-recognizers used the judgment scale differently. However, both groups show metacognitive awareness into the difficulty of a comparison. Confidence and rated difficulty moderately predicted accuracy. Judgments are therefore calibrated to one’s own accuracy to some extent (Gettleman et al., 2021; Weber & Brewer, 2004; Wixted & Wells, 2017). In the few studies which have tested this in face matching, they found moderate relationship between confidence and accuracy (Bruce et al., 1999; Stephens et al., 2017; White et al., 2014). These studies were conducted on the general population (non-face specialists). We show in for the first time this study that this moderate relationship extends to samples of both facial examiners and super-recognizers. In previous studies, metacognitive awareness in super-recognizers was assessed with questionnaires about broad face recognition ability (e.g., “I can spot familiar people in unexpected contexts.”). Their self-assessments were more accurate than the general population, but super-recognizers predicted their ability only at moderate or poor levels (Bate & Dudfield, 2019; Bobak et al., 2018). Therefore, this moderate degree of metacognitive awareness is observed for both self-assessed measures from previous studies and the implicit, decision-based measures from this study (average confidence and difficulty ratings). Recent work on the general population compared global and decision-based estimates of accuracy directly (Kramer et al., 2022). They showed that global assessments of ability may not be as informative as decision-based metrics, such as confidence on a trial. It would be useful to extend this evaluation for face specialist recruitment. Future work can directly compare the utility of self-reported estimates of ability from questionnaires and implicit, decision-based estimates of accuracy in face specialist populations to inform whether recruitment efforts can be served better by one over the other.

Regularly measuring accuracy and behaviors will clarify the effects of training and facial expertise. This practical, applied evaluation informs the underpinnings of facial expertise on a theoretical level. Different, and equally accurate, approaches supported high facial comparison accuracy in this challenging test. This approach deviates from other studies which measured behaviors associated with different levels of accuracy (Bobak et al., 2017; Hu et al., 2017; Norell et al., 2015; Tardif et al., 2018; Towler et al., 2017, 2021; White et al., 2015). Those findings showed accuracy in the general population can be predicted by different behaviors. Our work shows among face experts, multiple behaviors, and perhaps cognitive routes (Towler et al., 2021), lead to equally high levels accuracy.

Pivoting toward directly measuring behaviors of interest can lead to evidence-based development of standards and formalized procedures. Ultimately, a primary goal is to understand which behaviors are influenced by training or experience, and which are not. This will be answered with long-term experiments targeted toward separating the role of nature (natural ability), nurture (training/professional experience), and assessing their overlap. Researchers and practitioners need to continue working together to ensure the findings extend to real-world applications (Ramon et al., 2019).

## Supplementary Material

Supp1

## Data Availability

The de-identified data can be provided by the National Institute of Standards and Technology (NIST) by signing a data transfer agreement with NIST. Requests for the data should be submitted to Carina Hahn: carina.hahn@nist.gov. Code used for analyses and visualizations is available from: https://github.com/usnistgov/face-recognition-humans-machines/tree/main/Examiners%20vs.%20Super-recognizers.

## Footnotes

i See Figure S1 for a corresponding visualization for difficulty ratings.
