# Supplementary material for: Forensic facial examiners versus super-recognizers: Evaluating behavior beyond accuracy
Source: Appl Cogn Psychol. Author manuscript; Available in PMC 2024 Apr 26. (PMC11047140; doi:10.1002/acp.4003)
Supplement: Supp1 [file NIHMS1984568-supplement-Supp1.docx]

**Appendix:** **Supporting Information**


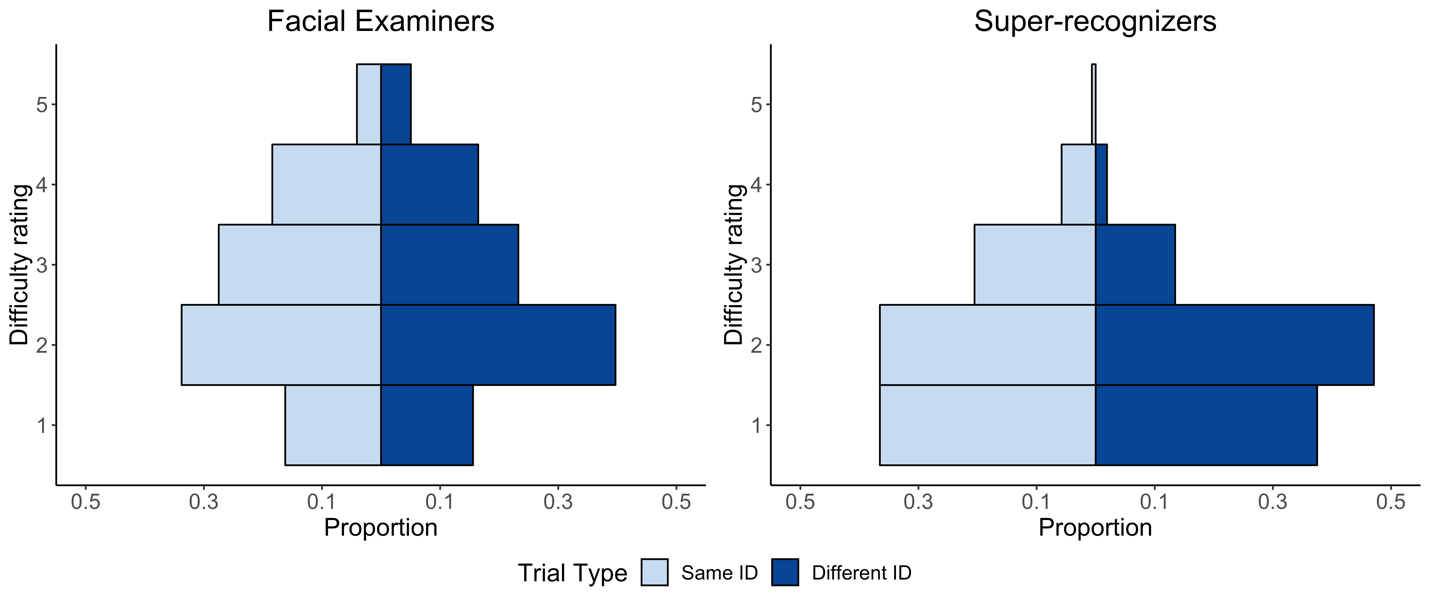


Figure S1. Back-to-back histograms depicting the proportion of judgments (x-axis) of a given difficulty rating (y-axis). Facial examiners are shown in the left-side graph; super-recognizers on the right-side graph. For each graph, the left, light blue side shows the response distributions for same-identity trials. The right, dark blue side shows the distributions for different-identity trials.


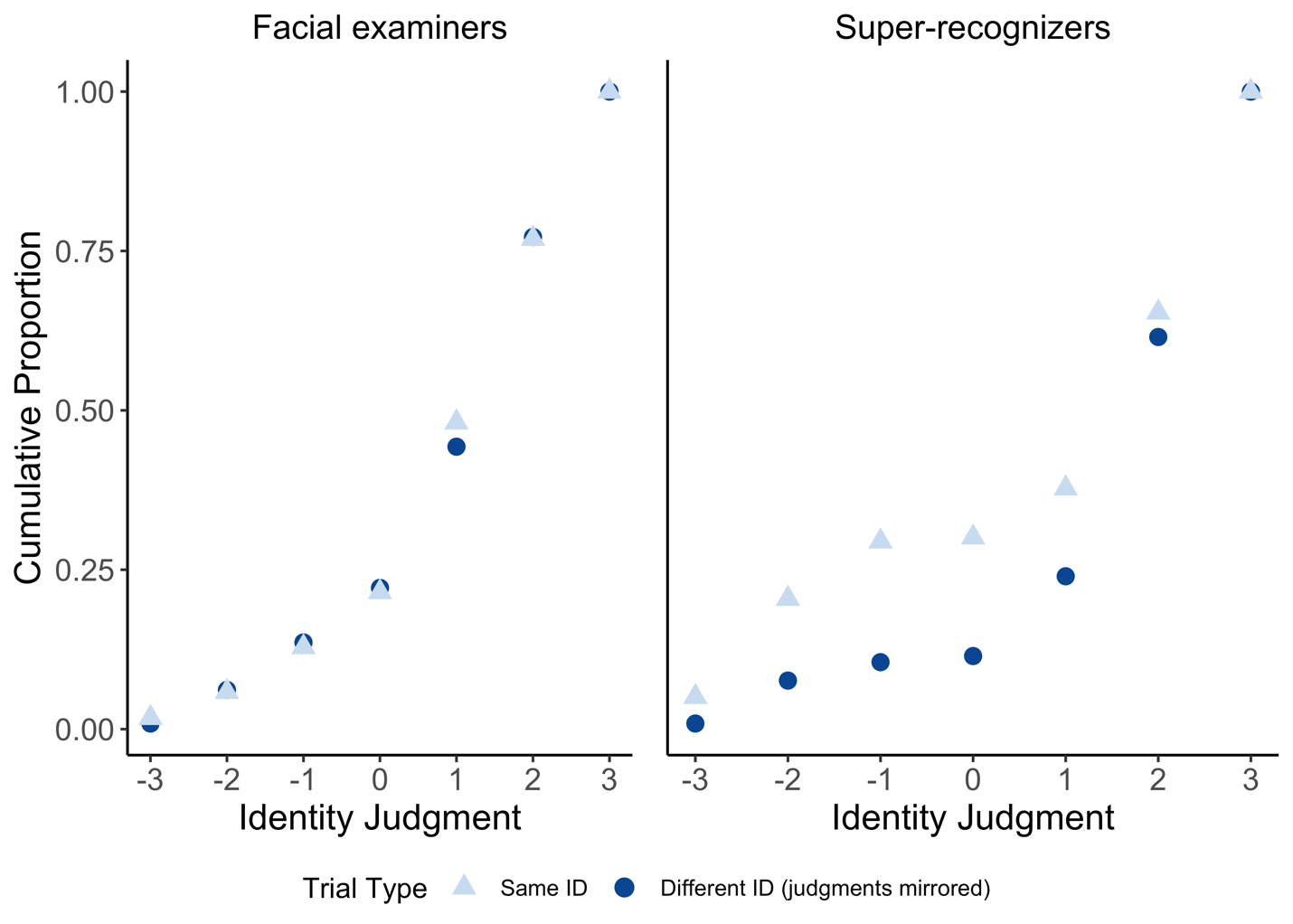


Figure S2. Cumulative response distributions of identity judgment responses. The x-axis shows the identity judgment scale. To allow judgments to be comparable across trial types, identity judgments for different identity trials are reversed for this visualization and corresponding analysis (e.g., responses of -3 are converted to +3, and so on). The y-axis shows the cumulative proportion of judgments. Point colors and shapes signify the trial type: light blue triangles show the cumulative proportion of judgments for same-identity trials; dark blue circles show the cumulative proportion of judgments for different-identity trials. Points for facial examiners almost completely overlap, indicating almost no difference in scale use for same and different-identity trials. For super-recognizers, judgments on same-identity trials spike sooner relative to different identity judgments. This reflects a more conservative approach to different-identities than same identities. See main text for analysis details.


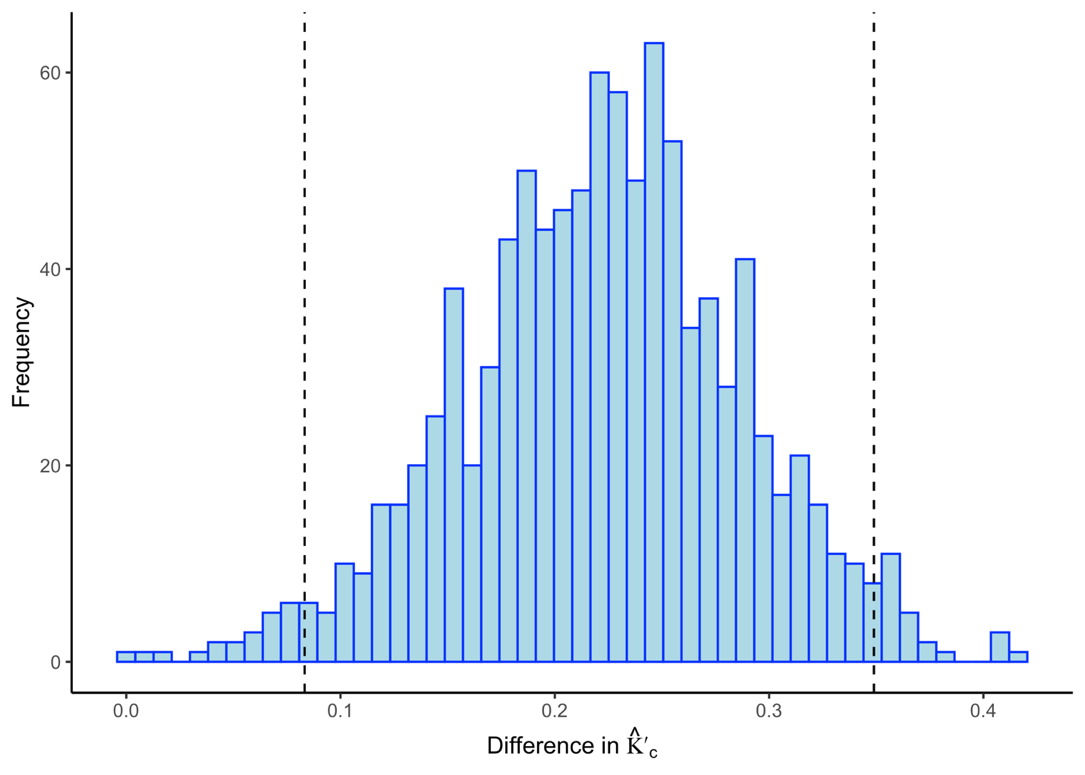


Figure S3. A histogram of the differences between pairwise facial examiner identity judgment agreement (${\hat{\boldsymbol{\kappa}}}_{\boldsymbol{c}}^{\boldsymbol{ʹ}}$)and super-recognizer ${\hat{\boldsymbol{\kappa}}}_{\boldsymbol{c}}^{\boldsymbol{ʹ}}$. This is the result of a bootstrap analysis with *n* = 1000 iterations. For each bootstrap iteration, image pairs were randomly selected with replacement. Identity judgment agreement (${\hat{\boldsymbol{\kappa}}}_{\boldsymbol{c}}^{\boldsymbol{ʹ}}$.) was measured based on those randomly selected images. We obtained the difference between facial examiner ${\hat{\boldsymbol{\kappa}}}_{\boldsymbol{c}}^{\boldsymbol{ʹ}}$ and super-recognizer ${\hat{\boldsymbol{\kappa}}}_{\boldsymbol{c}}^{\boldsymbol{ʹ}}$ (Difference in ${\hat{\boldsymbol{\kappa}}}_{\boldsymbol{c}}^{\boldsymbol{ʹ}}$ on the x-axis above). A value of 0 indicates no difference in agreement between the two groups. The higher the value, the greater the difference between their agreement levels. The histogram above depicts the differences obtained across all 1000 iterations. Dashed lines indicate the top and bottom 2.5% of the distribution which make up the 95% confidence interval [0.08, 0.35]. This indicates a significant difference in identity judgment agreement between the two groups.
